# Potentially Ineffective Care: Time for Earnest Reexamination

**DOI:** 10.1155/2014/134198

**Published:** 2014-04-06

**Authors:** William L. Jackson, Joseph F. Sales

**Affiliations:** ^1^Inova Health System, 8110 Gatehouse Road, Suite 600, West Tower, Falls Church, VA 22042, USA; ^2^Virginia Commonwealth University School of Medicine, Inova Fairfax Medical Campus, 3300 Gallows Road, Falls Church, VA 22042, USA

## Abstract

The rising costs and suboptimal quality throughout the American health care system continue to invite critical inquiry, and practice in the intensive care unit setting is no exception. Due to their relatively large impact, outcomes and costs in critical care are of significant interest to policymakers and health care administrators. Measurement of potentially ineffective care has been proposed as an outcome measure to evaluate critical care delivery, and the Patient Protection and Affordable Care Act affords the opportunity to reshape the care of the critically ill. Given the impetus of the PPACA, systematic formal measurement of potentially ineffective care and its clinical, economic, and societal impact merits timely reconsideration.

## 1. Brief Case Vignette

A 76-year-old woman with a history of chronic obstructive pulmonary disease (COPD), atherosclerotic coronary artery disease, renal cell carcinoma, and early dementia presented to the emergency department with obtundation, relative hypotension, and labored breathing. She was intubated for airway protection, central access was obtained, and she was subsequently admitted to the intensive care unit (ICU) with hypercapnic respiratory failure and suspected pneumonia.

She had been residing in an assisted living facility for six years prior to presentation, though she had required two hospital admissions and subsequent subacute rehabilitation stays for COPD exacerbations within the preceding six months. She was widowed, and her three children lived in other areas of the country. When contacted regarding her mother's ICU admission and advance directives, her eldest daughter remarked, “my mother is a pretty strong woman,” and “do what you have to do—she wants to live.”

Her initial hospital course was notable for vasopressor-dependent circulatory shock and progressive hypoxemia, but she was able to follow commands intermittently. Computed tomography pulmonary angiography and transthoracic echocardiography revealed no evidence of pulmonary embolism. Coronary artery catheterization was performed for moderately elevated troponin levels and newly noted systolic dysfunction and revealed nonobstructive disease. Acute renal failure developed and was managed with several sessions of intermittent hemodialysis. Despite shock resolution and appropriate therapy for COPD and health-care-associated pneumonia, the patient failed two extubation attempts and required tracheostomy on the nineteenth hospital day. A percutaneous cholecystostomy drain was placed for leukocytosis and biliary sludge. She was transferred to the stepdown unit during the fifth week of hospitalization.

With the exception of a rapid response call for hypotension that resulted in ICU transfer and four days of vasopressor support, her stepdown stay was relatively uneventful. While she was able to open her eyes on occasion, she did not manifest purposeful movements. Diagnostic evaluation for her depressed sensorium was unrevealing. Palliative care input and continued dialogue with her family about goals of care ultimately culminated in a “do not resuscitate” order, and she died on the seventy-second hospital day.

## 2. Introduction

National health spending in the United States has been expanding at a relentless and unsustainable rate, particularly when viewed relative to other segments of the economy [1]. While this spending growth spares few areas of the health care system, critical care medicine provides an abundant area for analysis, as its costs continue to increase both overall and as a percentage of US gross domestic product [2]. Accordingly, establishing a standard means for examining the cost effectiveness and value of critical care delivery should be a priority for public policy.

Just over fifteen years ago, Esserman and colleagues introduced the term* potentially ineffective care* (PIC) to describe care that exhibited a combination of high resource utilization and limited patient survival within the hospital or following hospital discharge [3]. Their work proposed a systematic approach using a refined severity of illness model to identify ICU patients at risk for high costs and early death. A contemporaneous publication from the SUPPORT project [4] highlighted considerable deficiencies in the applied value of prognostic information, patient-physician communication, and end-of-life therapy in the inpatient setting and further revealed the need to address inappropriately prolonged care. Given the findings of these two studies, there appeared to be considerable impetus and potential for developing robust means of responsibly improving preferential and economic aspects of ICU practice.

However, the possibility of a broad paradigm shift to integrate PIC-driven outcome measures into the delivery of quality ICU care was subsequently attenuated by several developments. While the cost constraints and slowed rate of expenditures brought on by the emergence of managed care appeared to lower the risk of PIC outcomes [5], the potential rationing implications behind this observation and the possibility of associated higher risk-adjusted mortality remained unexplained and unpalatable [6]. Scrutiny of macroeconomic assumptions about potential financial savings called into question the ability of the health care system and clinicians to simply achieve cost reduction from ICU management targeted at end-of-life care [7]. Several developments in the clinical setting also curbed significant further inquiry. First, there were acknowledged limitations in trying to apply population-based prognostic models to individual patients. Second, investigation was limited by industry migration away from the health maintenance organizations and capitated reimbursement plans that could have served as fertile arenas for study. And third, the recognized impotence of advanced directives and information sharing as significant drivers to lower PIC remained. What follows summarizes the vicissitudes of PIC in recent history and urges reinvigoration of the concept.

## 3. Advent and Implications of the Patient Protection and Affordable Care Act 

The revamping of the US health care system heralded by the Patient Protection and Affordable Care Act of 2010 (PPACA) might reasonably mark the reentry of PIC as a focal point for intervention. At risk of being lost amid the hyperbole in the lay press about end-of-life care and its subsequent omission from reform is the opportunity to address actual deficiencies in such care [4]. Furthermore, there is a pressing need to identify areas where the health care system should best apply scarce resources and where financial pressures are likely to influence organizational and individual behavior. It is possible, if not probable, that many of the same economic incentives observed in the ICU with managed care [6, 8] might emerge as PPACA initiatives are implemented, and the proposed structure of accountable care organizations (ACOs) [9] should recognize such incentives. Indeed, though much of the rationale behind PPACA centered around lowering the growth rate of health care spending, the ability of this legislation to favorably bend the cost curve may be limited [10]. Therefore, ancillary approaches to cost containment, particularly in resource-intensive areas such as the ICU, should be explored. Though recent experience in Massachusetts to reduce costs through legislation has been inconclusive, the magnitude of health care spending attributed to the ICU makes the necessity to address PIC even more pressing. Historical precedent adds context to the argument.

Among the most significant developments that affected the health care economy in the United States in the latter half of the twentieth century was the rise of managed care organizations as a means to deliver and pay for health care. Their genesis on a large scale can be traced to the 1930s, with Kaiser Permanente's use of vertical integrated group practices and financing through prepayment to efficiently provide affordable and comprehensive health care services to a growing industrial sector above and beyond caring for workplace injuries; this became known as the HMO model [11]. Economic viability was sustained by restricting access to providers within the group. A vigorous debate surrounding health care industry reform in the 1990s stimulated a renaissance in health care economics and a proliferation of theoretical models to address cost containment, while generating disparate assessments as to whether escalating costs were due to consumer demand problems or problems of health care resource supply. Prescriptions centered around regulatory controls, competition between insurers and providers, improved utilization management, and price and reimbursement controls. Managed care plans introduced an economic rationalism into the health care industry congenial to payors and providers that rewarded service providers performing more efficiently than marketplace competitors. Furthermore, this evolution fostered a process-oriented view to health care quality improvement that connected all the agents into a single delivery system and deployed novel metrics to evaluate performance of those systems. Indeed, it was in this environment that the number of individuals covered under managed care plans grew fivefold between 1980 and 1995 [12].

Nevertheless, the widespread discussion of health care in policy circles as a public good to be treated with tools of economics did little to address how savings could be gained in specific segments of the health care delivery system (such as the ICU) and how different modalities of treatment could be leveraged to achieve quality care while also preventing waste. Assessment of PIC may offer insight into such subtleties.

## 4. Clinical Value

Prognostic uncertainty and its influence on the subsequent course of intensive care have been long recognized [13], and many of the clinical elements that influence the mortality component of PIC remain in flux. Continued modifications in ICU design and process, the technological imperative, evolving inpatient demographics to an older, sicker population, and worsening shortages of critical care physicians and nurses require an increasing level of sophistication and engagement to improve ICU outcomes. Moreover, while the pragmatic approach to ICU care presently, and not unreasonably, involves continuous reassessment of prognosis and goals of care in light of the clinical course, clearly something is missing. The oft-heard whispered sentiments among ICU staff about their own advance directives (e.g., “I would never do this to my father”), the frequent incongruity between patient desires and physician actions [4], and the problem of moral distress among caregivers [14] all suggest an unsustainable tension and ongoing abdication of responsibility. Early and appropriate use of palliative care, promulgation of hardwired proactive and novel means of periodically engaging patients and families candidly about goals and expectations of care [15], appreciation of ICU readmissions as opportunities for frank rephrasing of expectations [3], refinement of predictive models [16, 17], revisiting the burden of proof about ICU value [18, 19], and legal and legislative clarity about end-of-life care should all be encouraged. However, formal measurement of PIC outcomes [3, 5, 20, 21] and thoughtful examination of outcome implications may raise the professional standard for humbly but honestly interacting with patients and families.

## 5. Economic Value

The resource utilization component of PIC has relatively clear implications for evaluation of provider and organizational practice [6, 19, 22]. However, reexamination of PIC reaches well beyond singular case and provider cost control. Industry changes catalyzed by PPACA will impact local operational strategy and resultant capital budgeting and staffing considerations for individual health care systems. For many organizations, for which high proportions of PIC compound the high costs of ICU facilities and labor, decisions about expenditures on critical care services are truly existential. Improved and transparent exploratory data analysis might give PIC a transformative weight that surpasses that presently sought through process, core, and performance measures. In addition, PIC assessment should inform and shape philosophy about an optimal and just national inpatient care infrastructure [2, 23], at a time when details about ACOs, evolving delivery designs, and new risk-sharing payment models are being refined. Tailoring efforts to examine the economic impact on the chronically critically ill population [24], measuring cost and mortality thresholds in isolation, and assessing impact on severity-adjusted mortality rates should all be performed in tandem.

These latter two points are of particular significance, because the relationship between resource utilization and patient outcomes is not straightforward, and the complexity inherent to ICU care will necessitate humility in interpreting PIC measures. Several aspects deserve comment. First, there is evidence, albeit retrospective, suggesting increased spending and treatment intensity may positively impact mortality, both for common inpatient conditions [25] and end-of-life status [26]. Second, ICU bed availability may represent a singular favorable influence on mortality requiring explicit acknowledgment in proposed care models [27, 28]. Third, it is likely that uninsured status deleteriously impacts access to ICU care and mortality, though PIC frequency might be decreased [29]. Fourth, certain elements of ICU care may disproportionately (and debatably) impact costs; mechanical ventilation is just one example [30, 31]. And fifth, given the vagaries of measurement and the considerable significance of public reporting of inpatient mortality rates [32, 33], indiscriminate or disproportionate attention to PIC risks erroneous interpretation of quality of care.

## 6. Societal Value

Perhaps most important, there must be a more deep-rooted social and professional inquiry about end-of-life issues that shapes patient preferences well before ICU arrival, when it appears opportunity to influence behavior is relatively limited [4]. The moral foundation that could form the rationale for a “collective individual restraint” [34] that is voluntary, consistent with self-evident values, and respectful of social justice and our shared humanity should be explored specifically in the context of PIC. The importance of emotional and religious integrity in assisting with the establishment of such restraint requires further study [35, 36]. While fruitful dialogue about sacrifice, self-interest, and opportunity cost would require formal acknowledgment of objective standards, such conversation occurs presently, in the form of familial discussions about suffering, capacity, and bequeathed assets. Moreover, the motivations for and potential repercussions of controversial proposals to restrict care [37] that could impact PIC measurement should be examined rigorously, perhaps as part of a formal revamping of care delivery around quantifiable value [38, 39]. Formation of an American entity analogous to the British National Institute for Health and Clinical Excellence (NICE) could facilitate such examination [40, 41]. Indeed, the possibility of an undesirable limitation of medical innovation should be considered, as physiologic states carrying a poor prognosis in our current setting might be treatable in the future; research and training constructs would need to be developed to address this. Continued development of prospective models to quantify futility and guide clinicians should be encouraged. Lastly, recognition that unbridled deference to patient autonomy has readily tangible consequences beyond simple resource allocation, as in the instance of antibiotic resistance, is an important step in this process [42].

## 7. Alternative (Aspirational) Vignette Excerpt

She had been residing in an assisted living facility for six years prior to presentation, though she had required two hospital admissions and subsequent subacute rehabilitation stays for COPD exacerbations within the preceding six months. She was widowed, and her three children lived in other areas of the country. When contacted regarding her mother's ICU admission and advance directives, her eldest daughter remarked, “my mother is a pretty strong woman and wants to live, but she has discussed her end-of-life wishes with us at length, and she would not want protracted efforts.” The intensivist subsequently gave a candid assessment of the patient's likely prognosis, drawing on the medical literature and her own practical experience, to develop a plan based on objective timelines and measures of clinical progress. Consistent with the patient's desires, early palliative care was initiated, the entire clinical care team was engaged in discussions regarding care, and the patient's pastor and extended family were invited to visit liberally. She subsequently died peacefully on hospital day number 4.

## 8. Conclusion

In sum, over the next several years, industry upheaval and economic pressures will mandate action to address the burgeoning costs of ICU care [43, 44], most of which is directed at some of the most vulnerable members of our society. Without an intentional effort to transparently identify the social value of certain end-of-life interventions, informed by academic debate and public deliberation, evolving economic incentives may result in mere cost-shifting and higher mortality in the name of eliminating suffering and advancing communal welfare. A timely reconsideration of PIC as a systematically measured outcome is indicated, so that “more proactive and forceful attempts at change” [4] do not result in unintended consequences, with potential harm to human dignity.

## General Issues with Potentially Ineffective Care

*Issues*

- Contribution to fractured, piecemeal care that may be divorced from societal and community implications [34],
- suboptimal and unsustainable value creation for health care system as a whole [38].

*Possible Actions*

- Development of national entities analogous to the British National Institute for Health and Clinical Excellence (NICE) [40, 41],
- continued promotion of clear-cut value in health care services [39],
- engagement of “societal shareholders”:
  - patients,
  - clinicians,
  - payers (health plans and government (CMS)),
  - public (clergy and advocacy groups).

## **Possible Approaches to Mitigate Potentially Ineffective Care in the Intensive Care Unit

*Issues*

- Incongruity between patient desires and physician actions [4],
- moral distress and impaired morale for ICU caregivers [14],
- ICU-specific interventions (e.g., mechanical ventilation [30, 31]) may disproportionately impact PIC-driven outcome measures.

*ICU-Specific Interventions*

- Early and appropriate use of palliative care,
- scheduled, proactive communication with patients and families [15],
- directed, appropriate conversation at time of ICU readmission [3],
- enhancement of predictive models [16, 17] and application of emerging and improving physiologic monitoring to prognostic science,
- limitation of ICU bed volumes and/or availability [27, 28].
